# Beyond lecanemab: Examining Phase III potential in Alzheimer's therapeutics

**DOI:** 10.1002/pcn5.185

**Published:** 2024-03-20

**Authors:** Hitoshi Osaka, Keiichiro Nishida, Tetsufumi Kanazawa

**Affiliations:** ^1^ Department of Neuropsychiatry Osaka Medical and Pharmaceutical University Takatsuki Osaka Japan

**Keywords:** Alzheimer's dementia, amyloid removal, lecanemab, therapeutic interventions

## Abstract

This review focuses on the development of therapeutic interventions for Alzheimer's dementia. While established treatments targeted acetylcholine and NMDA receptors, there is a growing demand for innovative therapies as the aging population increases. The paper highlights the US Food and Drug Administration's approval of aducanumab (Aduhelm) and lecanemab (Leqembi), emphasizing the developmental status of new treatments. Specifically, it covers seven principal drugs in Phase III trials, detailing their mechanisms of action, clinical trial specifics in the United States and Japan, and the current status of regulatory applications. The review focuses on amyloid removal (donanemab), tau protein mitigation (E2814), drug repositioning (Semaglutide, GV1001), and disease‐modifying small molecules (fosgonimeton, hydralazine, masitinib). However, Gantenerumab and Solanezumab, unsuccessful in Phase III, are not covered. While the future approval status remains uncertain, we hope these drugs will offer beneficial therapeutic effects for potential dementia patients.

## INTRODUCTION

The pursuit of therapeutic remedies for Alzheimer's disease (AD) stands as a formidable challenge. Established nations have long relied solely on medications targeting acetylcholine and NMDA receptors, leaving an exigent demand for more radical therapeutic avenues as the aging populace burgeons. The US Food and Drug Administration (FDA)'s sanction of aducanumab (Aduhelm; ostensibly eradicating amyloid‐β plaques in the brain) in 2021, assumes profound importance, given its potential impact on the multitude afflicted with AD. Moreover, with the recent full approval of lecanemab (Leqembi) in the United States and its ongoing submissions for approval in various nations, it becomes imperative to succinctly delineate the developmental status of forthcoming pharmaceutical candidates and their specific targets. In this exposition, we compile exhaustive data concerning principal drugs presently in Phase III trials across diverse geographic domains. This encompassing overview incorporates their putative mechanisms of action, affiliations with pharmaceutical enterprises and developmental laboratories, particulars of pivotal Phase I/II/III investigations, and the extant status of regulatory petitions.

Our collation is premised upon the recent analysis conducted by Cumming et al.,^1^ facilitating a nuanced comprehension of the subject matter. Cumming et al.'s comprehensive assessment employed an automated algorithm to scrutinize drugs aimed at AD and mild cognitive impairment (MCI) from ClinicalTrials.gov, aligning with the Common Alzheimer's Disease Research Ontology (CARO) and Disease Research Ontology (CADRO).^2^ Consequently, the comprehensive nature of the data remains contingent, given that not all drugs may possess scientifically validated mechanisms of action. The primary objective of this survey is to introduce the succeeding contender after lecanemab (Leqembi). The featured drugs' mechanisms of action are delineated, focusing on amyloid removal (donanemab) and tau protein mitigation (E2814), alongside pharmaceutical agents employing repositioning strategies (Semaglutide and GV1001) and modulating small molecules (fosgonimeton, hydralazine, masitinib). Consonant with the review's intent, Gantenerumab and Solanezumab, both unsuccessful in Phase III, were excluded. Inclusive of the entirety outlined in Table 1, Gantenerumab and Solanezumab are also encapsulated.

**Table 1:** Candidates for new dementia drugs.

Drugs in development	CADRO approach	Mechanisms	Subject characteristics	Current status of research (from: https://clinicals.gov)	Current status of research (from: PMDA in Japan)	Sponsor
Gantenerumab	Aβ	Anti‐amyloid monoclonal antibody direct at amyloid oligomers and plaque	Alzheimer's disease or MCI	US Phase II: 1 completed	N/A	MorphoSys and Hoffmann‐La Roche
				US Phase II/III: 1 completed		
				US Phase III: 7 completed		
				＊Phase IIItrials GRAUATE1 and 2 failed		
Solanezumab	Aβ	Anti‐amyloid monoclonal antibody direct at amyloid monomers	Mild to moderate Alzheimer's disease	US Phase I:１ completed	N/A	Eli Lilly and Company
				US Phase II: ２ completed		
				US Phase II/III: 1 completed		
				US Phase III: ６completed		
				＊PhaseIII trial A4 study failed		
Donanemab	Aβ	Anti‐amyloid monoclonal antibody specific for pyroglutamate plaque amyloid	MCI or mild Alzheimer's disease	US Phase II:２completed, 1 in progress	N/A	Eli Lilly and Company
				US Phase III: 1 completed, 4 in progress		
E2814	Tau	Anti‐tau monoclonal antibody	MCI or mild Alzheimer's disease	US Phase Ib/II: 1 in progress	N/A	Eisai Inc. and University College London
				US Phase II/III: 2 in progress		
GV1001 (Tertomotide)	Synaptic plasticity/neuroprotection	Human telomerase reverse transcriptase amyloid monomers	Mild to moderate Alzheimer's disease	US Phase II: 1 completed, 1 in progress	N/A	GemVax ＆Kael Inc.
		Transcriptase amyloid monomers	Alzheimer's disease	US Phase III: 1 in progress		
Fosgonimeton (ATH‐1017)	Synaptic plasticity/neuroprotection	Hepatocyte growth factor (HGF); activates signaling via the HGF/MET receptor system; promotes survival of neurons, enhances hippocampal synaptic plasticity	Mild to moderate Alzheimer's disease	US Phase II: 1 completed US Phase II/III: 2 in progress	N/A	Athira Pharma, Inc
Semaglutide	Metabolism and bioenergetics	GLP‐1 agonist; anti‐inflammatory and insulin sensitivity effects	MCI or mild Alzheimer's disease	US Phase III: 3 in progress	Japan Phase 3: 1 in progress	Novo Nordisk Inc.
Hydralazine	Oxidative stress	Free radical scavenger	Mild to moderate Alzheimer's disease	US Phase III: 1 in progress	N/A	Shahid Sadoughi University of Medical Science and Health Sciences
(hydrochloride)						
Masitinib	Inflammation	Tyrosine kinase inhibitor exhibits neuroprotection via inhibition of mast cell and microglia/macrophage activity	Mild to moderate Alzheimer's disease	US Phase II: 1 completed	N/A	AB Science
				US Phase III: 1 completed, 1 in progress		

*Note*: All information is based on year‐end 2023. Of the nine new candidates represented, seven are detailed in the main text. The remaining two candidate drugs (Gantenerumab and Solanezumab) were dropped in Phase III and are therefore only noted in this table. CADRO, Common Alzheimer's and Related Dementias Research Ontology) https://iadrp.nia.nih.gov/about/cadro; MCI, mild cognitive impairment.

Albeit the future approval status of dementia therapeutics remains indeterminate, the envisioned prospect entails these seven drugs heralding a substantial therapeutic impact, offering solace to myriad dementia patients.

## AMYLOID REMOVAL

### Donanemab

Donanemab, a recombinant monoclonal antibody targeting N‐terminal pyroglutamylated amyloid‐β (N3pG Aβ), is a humanized antibody specifically directed against the N‐truncated pyroglutamylated amyloid‐β peptide at position 3 (pGlu3‐Aβ, AβpE3), currently under investigation as an AD treatment.^3^ Strategies to address pGlu3‐Aβ involve reducing its formation via glutamyl cyclase (QC), which catalyzes N‐cleaved Aβ to form pGlu3‐Aβ, along with the use of anti‐pGlu3‐Aβ antibodies.^4^, ^5^ Donanemab‐incorporated antibodies aim to eliminate pGlu‐Aβ postformation and/or hinder aggregation. Notably, donanemab exhibits potent efficacy against amyloid plaques, particularly nucleated plaques in the central nervous system.^6^ Its development is spearheaded by Lilly and Company.^7^

In the Phase II TRAILBLAZER‐ALZ trial, donanemab was administered at 700 mg for the initial three doses, followed by 1400 mg every 4 weeks up to 72 weeks, with blinded dose reduction assessments conducted at Weeks 24 and 52. Notably, the rate of donanemab‐induced amyloid reduction at 24 weeks exhibited a modest correlation with baseline amyloid levels. Analysis also indicated a delay in tau deposition compared with placebo, showcasing a significant 34% reduction in total tau levels with donanemab.^8^

In the subsequent TRAILBLAZER‐ALZ 2 study, a randomized, double‐blind, placebo‐controlled trial, participants received intravenous donanemab every 4 weeks or placebo for 72 weeks. Donanemab was administered intravenously at 700 mg for the first three doses and then 1400 mg every 4 weeks for up to 72 weeks. Those on donanemab were switched to placebo in a blinded manner upon meeting dose completion criteria. The primary outcome assessed changes in Integrated Alzheimer's Disease Rating Scale (iADRS) scores over 76 weeks, revealing a substantial delay in clinical deterioration, especially in individuals with mild to moderate tau accumulation and early‐stage amyloid‐positive AD.^9^

Safety evaluations demonstrated favorable tolerability, with mild to moderate injection reactions being the most common adverse events.^10^ Eli Lilly Japan K.K. has completed the filing of donanemab in Japan for the treatment of MCI and mild dementia due to AD, furthering its potential clinical reach.^11^

## TAU PROTEIN REMOVAL

### E2814

E2814, an anti‐tau monoclonal antibody, targets the aggregation and fibrillation of tau protein, pivotal pathological elements in AD characterized by neurofibrillary tangle formation. Inhibiting the in vivo degradation of fragments containing microtubule‐binding regions (MTBRs) of tau protein, E2814 curtails the spread of tau lesions across various brain regions. Developed as a therapeutic agent for tauopathies, its focus lies in impeding further neurofibrillary tangle accumulation, thereby acting as a disease modifier. Collaborative efforts between Eisai and University College London brought forth E2814, showcasing promise not only in tauopathies but also in addressing AD.^12^, ^13^

Clinical investigations into E2814 include an open‐label Phase Ib/II study evaluating safety and target engagement in individuals with mild to moderate cognitive impairment stemming from dominantly inherited AD (DIAD).^14^ Additionally, an ongoing open‐label Phase IIb/II trial, conducted by St. Louis Washington University School of Medicine, is dedicated to exploring E2814's efficacy in DIAD. The Dominantly Inherited Alzheimer Network Trials Unit (DIAN‐TU) spearheads the TauNexGen trial, a Phase II/III initiative aimed at assessing the safety, tolerability, biomarkers, and cognitive effects of this anti‐tau investigational drug in symptomatic and asymptomatic individuals harboring AD‐causing genetic mutations.^15^

The study design involves symptomatic participants receiving either the anti‐amyloid‐β (Aβ) protofibril antibody lecanemab for 6 months followed by treatment with the anti‐MTBR tau antibody E2814 or a placebo through randomized allocation. Evaluating the effectiveness of anti‐tau therapies by initially removing Aβ and examining subsequent outcomes in AD follows the temporal sequence of Aβ accumulation preceding tau in the brain. Asymptomatic individuals will be randomized to receive either the anti‐tau medication or placebo, with lecanemab administered to both groups after a year of treatment. This staggered administration aims to discern the impact of individual anti‐tau medication prior to evaluating combined drug effects. Should primary and secondary endpoints be met within 2 years of study initiation, an extension for an additional 2 years is anticipated for further analysis.^16^

## SYNAPTIC PLASTICITY/NEUROPROTECTION

### GV1001

GV1001, a vaccine peptide, targets a specific peptide fragment of telomerase reverse transcriptase to stimulate the immune system.^17^ Initially designed as an anticancer vaccine for pancreatic cancer,^18^ it is currently undergoing testing for AD. GV1001 represents a portion of the human enzyme telomerase reverse transcriptase, possessing antioxidant, antiapoptotic, and cell survival‐promoting properties. In cellular systems, tertomotide, a component of GV1001, exhibited characteristics akin to full‐length telomerase reverse transcriptase, mitigating Aβ toxicity and displaying anti‐inflammatory and antioxidant traits.^19^ GemVax & Kael, based in Korea, leads the development of GV1001.^20^

A Phase II multicenter clinical trial aimed to assess the safety and efficacy of GV1001 in mild to moderate AD patients. Participants received either 0.56 or 1.12 mg of the drug weekly for 4 weeks, followed by 10 injections every 2 weeks until Week 24. The findings indicated good tolerability, with statistical significance in efficacy observed only with the higher concentration (1.12 mg) as measured by the Clinical Dementia Rating Box‐Summary (CDR‐SB) and Alzheimer's Disease Cooperative Study‐Activities of Daily Living (ADCS‐ADL).^21^ Common adverse effects included nasopharyngitis, diarrhea, anxiety, back pain, arthralgia, and loss of appetite, occurring at an incidence rate of less than 10%.^21^

In Korea, an endorsed Phase III clinical trial for AD has been initiated,^22^ while a Phase II clinical trial for AD has commenced in the United States.^22^

### Fosgonimeton (ATH‐1017)

Fosgonimeton (ATH‐1017) is a small compound developed to enhance the activity of hepatocyte growth factor (HGF) and its receptor, mesenchymal‐epithelial transition factor (MET).^23^ This amplification of HGF/MET, a critical neurotrophic signaling pathway, holds potential to bolster neuronal health and function, potentially mitigating neurodegeneration in dementia.^24^ Evidence supports fosgonimeton's neuroprotective properties, notably its ability to significantly enhance cell survival against various neurotoxicities like MPP+, glutamate, lipopolysaccharides‐induced cytotoxicity, and oxidative stress in cortical neurons treated with HGF/MET positive modulators. Additionally, it is believed to potentially alleviate mitochondrial dysfunction associated with neurodegeneration and increase mitochondrial membrane potential.^23^

Athira Pharma, Inc. is currently developing the first prodrug formulation of this small molecule discovered through a screen for positive regulators of HGF/MET signaling. Fosgonimeton can be administered via a simple at‐home subcutaneous injection, akin to insulin injections. In a Phase I clinical trial involving 88 subjects receiving once‐daily subcutaneous injections, the safety, tolerability, pharmacokinetics, and pharmacodynamics of fosgonimeton were evaluated. Results indicated its safety and good tolerability across all dosages, showing a notable impact on ERP P300 latency normalization in AD patients compared with placebo (*p* = 0.027; *n* = 7 with 40 mg fosgonimeton and *n* = 4 with placebo).^25^

The 26‐week Phase II accelerate cure/treatments for all dementias (ACT‐AD) trial, a randomized, double‐blind, placebo‐controlled study in mild to moderate AD patients, examined the effects of fosgonimeton. Patients received subcutaneous fosgonimeton at either 40 or 70 mg once daily or a placebo. The primary endpoint of change in the biomarker ERP P300 latency was not statistically significant for the entire study population. However, a prespecified subgroup analysis of patients receiving fosgonimeton monotherapy displayed improvements in ERP P300 latency and Alzheimer's Disease Assessment Scale (ADAS)‐Cog11 at Week 26 compared with placebo, demonstrating pharmacological activity. The drug exhibited a generally favorable safety profile with no treatment‐emergent serious adverse events or deaths observed, while injection site reactions were the most common adverse events.^26^, ^27^

Currently, fosgonimeton is being evaluated in the late‐stage LIFT‐AD trial, a randomized, double‐blind, placebo‐controlled study designed to assess its safety and efficacy in mild to moderate AD patients not concurrently using acetylcholinesterase inhibitors. The trial is ongoing with additional patients being enrolled.^28^

## OTHERS

### Semaglutide

Oral semaglutide, a glucagon‐like peptide‐1 receptor agonist (GLP‐1 RA) drug, received FDA approval for treating type 2 diabetes in 2017.^29^ It boasts a well‐established safety profile.^30^, ^31^ GLP‐1, a hormone synthesized in the gastrointestinal tract, stimulates receptors in the gut, liver, and pancreas, triggering insulin release and restoring insulin sensitivity.^32^ Notably, insulin resistance in the brain is observed among some AD patients, prompting the investigation of semaglutide as a potential AD medication. Moreover, semaglutide's multifaceted impact on various cell types, including neurons, suggests a potential enhancement in neuronal, inflammatory, and vascular health, potentially slowing the clinical progression of AD.^33^, ^34^, ^35^ Research also indicates the involvement of brain GLP‐1 receptors in learning and neuroprotection.^36^

An epidemiological study revealed that diabetic patients treated with semaglutide demonstrated a reduced likelihood of developing dementia compared with those receiving other antidiabetic medications. This study combined data from a randomized, double‐blind, placebo‐controlled trial and information on GLP‐1 RA exposure and subsequent dementia diagnosis in type 2 diabetes patients, integrating findings from three randomized, double‐blind, placebo‐controlled cardiovascular outcomes trials involving 15,820 patients. The rates of dementia in patients randomized to GLP‐1 RA were significantly lower than those randomized to placebo (hazard ratio [HR]: 0.47 [95% confidence interval [CI]: 0.25–0.86]). Consistency in results was observed across national cohorts (HR: 0.89, 95% CI: 0.86). These findings suggest that GLP‐1 RA treatment is linked to a reduced dementia risk in type 2 diabetes patients, potentially decreasing its incidence.^37^

Two placebo‐controlled Phase III trials, EVOKE and EVOKE Plus, are underway to explore the efficacy of 14 mg oral semaglutide in individuals with early‐stage AD.^38^, ^39^, ^40^ Each study will enroll 1840 individuals aged 55–85 years with MCI due to AD or mild AD dementia who test positive for amyloid. Participants will be randomly assigned in a 1:1 ratio to receive either oral semaglutide at a 14‐mg dose once daily or a placebo for 156 weeks. The primary endpoint will be the change in the CDR‐SB score from baseline to Week 104, with secondary endpoints including changes in the MCI score and time to dementia progression in individuals diagnosed with MCI at baseline. Common side‐effects associated with semaglutide include nausea, diarrhea, and vomiting.^30^

### Hydralazine (hydrochloride)

Hydralazine, a vasodilator acting directly on vascular smooth muscle, is primarily used in treating hypertension.^41^ Oxidative damage to lipids and misfolding of amyloid beta (Aβ) contribute significantly to AD pathogenesis. Leveraging its role as a smooth muscle relaxant utilized in hypertension treatment, hydralazine is considered a potential candidate for AD pharmacotherapy due to its ability to reduce Aβ production and prevent oxidative damage via its hydrazide group with antioxidant properties.^42^, ^43^

Furthermore, studies have highlighted hydralazine's capacity to diminish neuroinflammation and oxidative stress damage, improve mitochondrial function, downregulate pro‐inflammatory gene expression, and upregulate antioxidant gene expression. Administering hydralazine during the early stages of AD may offer benefits by impeding pathological progression through the inhibition of neuroinflammation and oxidative stress.^43^

Currently, the Shahid Sadoughi University of Medical and Health Sciences is conducting a Phase III trial evaluating hydralazine. This placebo‐controlled trial will enroll 424 participants clinically diagnosed with mild to moderate AD. Participants will receive either 75 mg of hydralazine (25 mg thrice daily) or placebo. The primary outcome will assess the progression of AD using the ADAS at Months 3, 6, 9, and 12.^44^

However, adverse events associated with hydralazine encompass serious reactions with varying frequencies, including systemic lupus erythematosus‐like symptoms (fever, erythema, arthralgia, chest pain), fulminant hepatitis, hepatitis, liver dysfunction, jaundice, congestive heart failure, induction of angina, paralytic ileus, respiratory distress, acute renal failure, hemolytic anemia, pancytopenia, polyneuritis, and vasculitis.^45^

### Masitinib

Masitinib, a first‐in‐class oral kinase inhibitor, uniquely targets mast cells and macrophages/microglia. It has garnered approval for treating amyotrophic lateral sclerosis, slowly progressive systemic mastocytosis, severe asthma, progressive multiple sclerosis, and first‐line pancreatic cancer with pain.^46^

In diverse Phase IIB/III trials, masitinib has shown promising outcomes across various conditions, including amyotrophic lateral sclerosis, systemic mastocytosis, severe asthma, multiple sclerosis, first‐line pancreatic cancer, AD, and metastatic castration‐resistant prostate cancer. It specifically inhibits c‐kit on mast cells and exhibits neuroprotective properties in AD while targeting Fyn kinase.^47^

Under development by AB Science (France), the Phase II study for AD demonstrated significantly slower clinically relevant cognitive decline (≥4 point increase) measured by ADAS‐Cog response after 12 and 24 weeks with masitinib 4.5 mg/kg/day compared with placebo (*p* = 0.040 and *p* = 0.046).^48^

Moreover, results from the Phase III trial indicated a notable cognitive improvement with masitinib (4.5 mg/kg/day) versus placebo. The ADAS‐Cog primary endpoint reflected an improvement of −1.46 (95% CI [−2.46, −0.45]) versus 0.69 (95% CI [−0.36, 1.75]) in favor of masitinib, representing a significant difference between groups (−2.15; 97.5% CI [−3.48, −0.81]), *p* < 0.001.^49^

AB Science has received approval to initiate a confirmatory Phase III study enrolling 600 patients diagnosed with mild to moderate AD, evaluating masitinib's efficacy using the ADCS‐ADL score and Mini‐Mental State Examination (MMSE) score between 14 and 25.^50^

Although adverse events were more frequent with masitinib treatment (65% vs. 38% of patients), most were mild or moderate and transient. Serious adverse events occurred with similar frequency in both masitinib and placebo groups (15% vs. 13% of patients, respectively), with masitinib‐related events including gastrointestinal disturbances, edema, and rash.^49^

## DISCUSSION

This paper serves as a continuation of our previous review concerning forthcoming antipsychotic and antidepressant drugs,^51^ aiming to furnish contemporary psychiatric clinicians with updated pharmacological insights and to invigorate further research in this domain.

The percentage of the population aged 65 and older is increasing in almost every country, and according to the World Health Organization, as of September 2022, approximately 55 million people worldwide had dementia. This number is projected to increase to 78 million by 2030 and 139 million by 2050. The dementia drugs market is estimated to be worth US$16.44 billion in 2024 and is projected to reach US$22.21 billion by 2029.^52^ Therefore, it is important to provide clinicians with a bird's eye view of the next generation of candidate drugs for dementia, which is also why this paper was written.

Lecanemab encounters three principal practical challenges: patient selection, side‐effects, and pricing. The precise identification of suitable candidates for lecanemab, confined to MCI, necessitates mandatory positron emission tomography or cerebrospinal fluid scans. Anticipating a price similar to aducanumab (Aduhelm), high costs might impede widespread utilization.

Donanemab, which has a similar mechanism of action of amyloid removal, has been associated with the severely controlled adverse reactions overall ARIA‐E (amyloid‐related imaging abnormalities‐edema), ARIA‐H (amyloid‐related imaging abnormalities‐ cerebral microhemorrhages), which should be strictly controlled, was 24.0% and 19.7%, respectively, compared with 12.5% and 17.0% for lecanemab, respectively, and the incidence was not reduced.

Therefore, posttreatment magnetic resonance imaging will continue to be important for follow‐up. The high cost of lecanemab is a function of many factors, and it is not known at this time how much it will be with donanemab. The potential for specific side‐effects or other factors linked to the mechanisms of action of the seven subsequent drugs could impede their launch and widespread adoption.

As for the pharmacologic action, aducanumab is a monoclonal antibody that targets the amyloid beta (Aβ) plaques associated with AD. Amyloid‐β accumulates in the brain in AD and is believed to cause neuronal dysfunction and death. Aducanumab aims to slow disease progression by identifying and eliminating these plaques.^53^

Like aducanumab, lecanemab is a monoclonal antibody that targets amyloid‐β plaques, but the form of amyloid‐β it targets is different. Lecanemab specifically acts on the soluble oligomeric form of amyloid‐β and is believed to slow the progression of AD by eliminating it.^54^

Donanemab, introduced here, is another monoclonal antibody that targets amyloid‐β plaques in AD, but donanemab targets a specific modified form of amyloid‐β, namely pyridine‐soluble β‐amyloid. This particular form of amyloid beta is thought to be strongly associated with the progression of AD.^9^

Beyond pharmacotherapy, dementia treatment encompasses psychotherapy, behavioral therapy, and varied cognitive rehabilitation methods. Additionally, conditions resembling dementia, such as normal pressure hydrocephalus, encephalitis, and depression, possess their own effective treatments, underscoring the crucial need for accurate diagnosis. Our aspiration is for the global population to increasingly benefit from advancements in dementia treatment.

## DISCLOSURE STATEMENT

Tetsufumi Kanazawa and Keiichiro Nishida are Editorial Board members of *Psychiatry and Clinical Neurosciences Reports* and a co‐authors of this article. To minimize bias, they were excluded from all editorial decision‐making related to the acceptance of this article for publication. The remaining author declares no conflict of interest.

## AUTHOR CONTRIBUTIONS

All of us made substantial contributions to the study concept, drafted the manuscript, approved the final version of the manuscript to be published, and agreed to be accountable.

## ETHICS APPROVAL STATEMENT

N/A

## PATIENT CONSENT STATEMENT

N/A

## CLINICAL TRIAL REGISTRATION

N/A

## Data Availability

N/A
